# Applicability of Multi-Sensor and Multi-Geometry SAR Data for Landslide Detection in Southwestern China: A Case Study of Qijiang, Chongqing

**DOI:** 10.3390/s25144324

**Published:** 2025-07-10

**Authors:** Haiyan Wang, Xiaoting Liu, Guangcai Feng, Pengfei Liu, Wei Li, Shangwei Liu, Weiming Liao

**Affiliations:** 1 Chongqing 208 Geological Environment Research Institute Co., Ltd., Chongqing 400700, China; wanghaiyancs@163.com (H.W.); liwei_cq@163.com (W.L.); 2 School of Geosciences and Info-Physics, Central South University, Changsha 410083, China; liuxting@csu.edu.cn; 3 Chongqing Institute of Geological Environment Monitoring, Chongqing 401147, China; liupf616@163.com (P.L.); shwwyy115@126.com (S.L.); 4 Chongqing Institute of Geology and Mineral Resources, Chongqing 401120, China; lwm0419@163.com

**Keywords:** landslide identification, LUTAN-1, L-band SAR, Southwest China’s mountainous areas

## Abstract

The southwestern mountainous region of China (SMRC), characterized by complex geological environments, experiences frequent landslide disasters that pose significant threats to local residents. This study focuses on the Qijiang District of Chongqing, where we conduct a systematic evaluation of wavelength and observation geometry effects on InSAR-based landslide monitoring. Utilizing multi-sensor SAR imagery (Sentinel-1 C-band, ALOS-2 L-band, and LUTAN-1 L-band) acquired between 2018 and 2025, we integrate time-series InSAR analysis with geological records, high-resolution topographic data, and field investigation findings to assess representative landslide-susceptible zones in the Qijiang District. The results indicate the following: (1) L-band SAR data demonstrates superior monitoring precision compared to C-band SAR data in the SMRC; (2) the combined use of LUTAN-1 ascending/descending orbits significantly improved spatial accuracy and detection completeness in complex landscapes; (3) multi-source data fusion effectively mitigated limitations of single SAR systems, enhancing identification of small- to medium-scale landslides. This study provides critical technical support for multi-source landslide monitoring and early warning systems in Southwest China while demonstrating the applicability of China’s SAR satellites for geohazard applications.

## 1. Introduction

The southwestern mountainous region of China (SMRC) is characterized by rugged terrain and complex geological structures, which contribute to frequent landslides occurrences. These slope failures pose significant threats to local communities, infrastructure, and socioeconomic development [1,2]. The Chongqing municipality, located in the core of this geologically vulnerable region, ranks among the most geohazard-prone areas in China, with high-risk zones covering 20.81% of its territory. As a key area for geohazard prevention and control in Chongqing, the Qijiang District experiences a large number of geohazards, with landslides being particularly prominent. Current records indicate these disasters threaten 3475 households (12,663 residents), with a potential economic loss of approximately 550 million RMB. In 2015, due to heavy rainfall, multiple landslides occurred across the district, severely affecting seven streets, including Zhuantang and Yongcheng. The disasters influenced the life of 2398 people and caused direct economic losses exceeding 1.66 million RMB. Therefore, conducting long-term, wide-area landslide monitoring in southwestern China’s complex mountainous terrain not only holds significant practical value but also presents higher technical demands for early warning and prevention of geohazards.

Conventional optical remote sensing techniques are often constrained by weather conditions, like cloud cover, limiting their capability for continuous landslide monitoring [3,4]. Synthetic Aperture Radar (SAR) technology has emerged as a vital tool for landslide detection in mountainous regions due to its all-weather and day–night observation capacity since 2000 [5,6,7]. However, different SAR sensors exhibit significant performance variations (e.g., Sentinel-1 versus LUTAN-1) in landslide identification, attributable to differences in wavelength, spatial resolution, and viewing geometry [8,9,10,11]. While Sentinel-1 data have been applied and well-studied in SMRC [12,13,14], the potential of China′s newly launched LUTAN-1 (LT-1) satellite remains underexplored, particularly the capability for landslide monitoring in vegetated areas and adaptability to complex terrains. Therefore, a systematic evaluation of multi-source SAR data performance in landslide detection in SMRC is necessary. On this basis, optimized landslide detection methodologies for mountainous regions can be established.

Recent advancements in InSAR technology have significantly enhanced landslide monitoring and detection. C-band Sentinel-1 data are widely used in SMRC due to their high temporal resolution and wide coverage. For example, Ran et al. detected 115 active landslides in Mao County using Sentinel-1 data [15], and Li et al. [16] identified 30 landslides in the Lancang River Basin using Sentinel-1 ascending and descending data. These studies confirm the applicability of C-band SAR data in mountainous, vegetated areas. However, C-band SAR data are prone to decorrelation in complex environments, resulting in discontinuous deformation signals and limited detection accuracy [17,18]. Therefore, researchers have begun to explore landslide detection using L-band SAR data. For instance, Shi et al. [19] identified 30 active landslides in the Three Gorges area using ALOS data, and Cao et al. [20] compared ALOS-2/PALSAR and Sentinel-1 data, revealing differences in detection performance. Although studies have confirmed the advantages of L-band SAR data in complex terrain and densely vegetated areas, ALOS-1/2 data suffer from low revisit frequency in most parts of China. LuTan-1, China’s L-band satellite, showed higher coherence and better deformation sensitivity in Mao County [21], but its systematic application in landslide detection in southwestern China remains limited.

Recently, researchers have increasingly focused on multi-source SAR data fusion strategies. Wang et al. [22] demonstrated that the combined use of ALOS-2/PALSAR and Sentinel-1 data significantly enhanced the landslide detection accuracy and completeness in the Niulan River Basin. Their results confirmed that multi-source SAR data fusion can effectively improve detection performance and mitigate the limitations of spatiotemporal resolution inherent in single-source datasets. However, most existing studies have not fully addressed the impact of multi-geometry on detection accuracy, and the effectiveness of data fusion remains limited by ALOS-2 SAR data’s low temporal resolution.

Despite progresses, InSAR technology faces several critical challenges in landslide monitoring and identification in SMRC. Sentinel-1 data show limited monitoring accuracy in vegetated areas, whereas ALOS-2 data suffer from low temporal resolution and high acquisition costs. The applicability of LT-1 data requires comprehensive validation. The impacts of different sensors and viewing geometries on landslide monitoring lack systematic analysis. Addressing these problems, this study takes the landslides in Qijiang, Chongqing, as a case study. Through integrated analysis of multi-source SAR data (Sentinel-1, ALOS-2, and LT-1) with ascending/descending observations, we aim to do the following: (i) evaluate performance of different band SAR data under complex topographic conditions, (ii) elucidate the influences of viewing geometry on identification accuracy, and (iii) develop optimal multi-sensor fusion strategies. The findings will provide technical support for enhancing the disaster monitoring capabilities of China’s satellites and establish a scientific basis for precise landslide identification in SMRC.

## 2. Study Area and Data

### 2.1. Study Area

**Figure 1:**
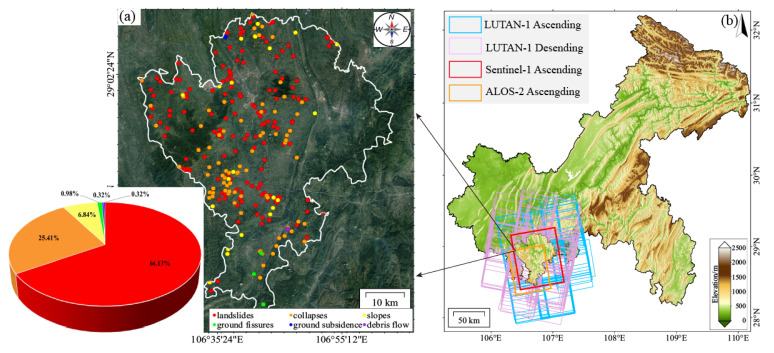
Overview of the study area. (**a**) Spatial distribution of recorded geological hazards in the study area with proportional classification; (**b**) topographic map and SAR image coverage of Qijiang District, Chongqing.

The Qijiang District (28°27′–29°11′ N, 106°23′–106°55′ E) is situated in southern Chongqing Municipality, China, at the transitional zone between the Sichuan Basin and the Yunnan–Guizhou Plateau (Figure 1a). The area features complex geological structures, where the northern extension of the Dalou Mountains intersects with the Huayingshan fan-shaped tectonic system. Encompassing 2182.14 km^2^, the terrain exhibits higher elevations along the periphery and lower elevations in the central basin, with an average altitude of 254.8 m (range: 18–1973 m). Mountainous areas account for 67.6% of the total area, while hilly regions constitute the remaining 32.4% [23].

The region experiences a typical subtropical monsoon climate, characterized by a mean annual temperature of 17.7 °C and an average annual precipitation of 1115.1 mm, with 70–80% of rainfall concentrated between May and October [24]. Intensive hydrological erosion coupled with frequent extreme weather events (e.g., torrential rains and prolonged rainy periods) has led to frequent occurrence of geological hazards.

The Qijiang District had documented 307 potential geological hazard sites as of June 2022. These include 203 landslides (66.13%), 78 collapses (25.41%), 21 unstable slopes (6.84%), and minor occurrences of ground fissures, subsidence, and debris flows (collectively 2.3%) [25]. Stability assessments reveal that 24.15% of these sites exhibit poor stability, while 64.15% remain stable. Landslides represent the primary geological risk source in this region (Figure 1a).

### 2.2. SAR and Auxiliary Datasets

To monitor surface deformation in Qijiang, we collected Sentinel-1 ascending data (102 scenes, 2018–2019 and 2023–2025), ALOS-2 ascending data (9 scenes, including 20180410/20180522/20180703/20180814/20180925/20190730/20190813/20190924/20191217), and LT-1 ascending/descending data (114 scenes, 2023–2025). Sentinel-1 (1 track, 1 frame) and LT-1 (2 tracks, 6 frame) provide full coverage of the study area, while ALOS-2 (1 track, 1 frame) only partially covers the region. A 30 m resolution SRTM DEM was applied for topographic phase correction. Detailed parameters of these datasets are listed in Table 1, and their spatial coverage is illustrated in Figure 1b.

## 3. InSAR Data Processing and Result Analysis

### 3.1. InSAR Data Processing

This study employed the multi-master SBAS-InSAR technique for processing Sentinel-1 and ALOS-2 data [26]. Given the relatively limited dataset of LT-1, Stacking-InSAR was adopted for its analysis.

The SBAS-InSAR processing was conducted following these key steps: The optimal master image was first identified through comprehensive evaluation of spatiotemporal baselines and baseline frequency distributions. Interferometric pairs were then carefully selected using predefined thresholds (perpendicular baseline B⟂ < 200 m; temporal baseline Δt < 100 days), followed by removal of topographic phases using a 30 m resolution DEM. The interferogram pairs subsequently underwent a multilooking operation with a 10:2 (range: azimuth) ratio. Phase unwrapping was then performed using the Minimum Cost Flow (MCF) algorithm, after which high-coherence points (γ > 0.7) were identified based on coherence, intensity, and amplitude dispersion criteria. In the final stage, orbital errors were first eliminated through quadratic polynomial surface fitting. Time-series analysis was then conducted using singular value decomposition (SVD), while nonlinear deformation and atmospheric phases were systematically separated from the residuals.

**Figure 2:**
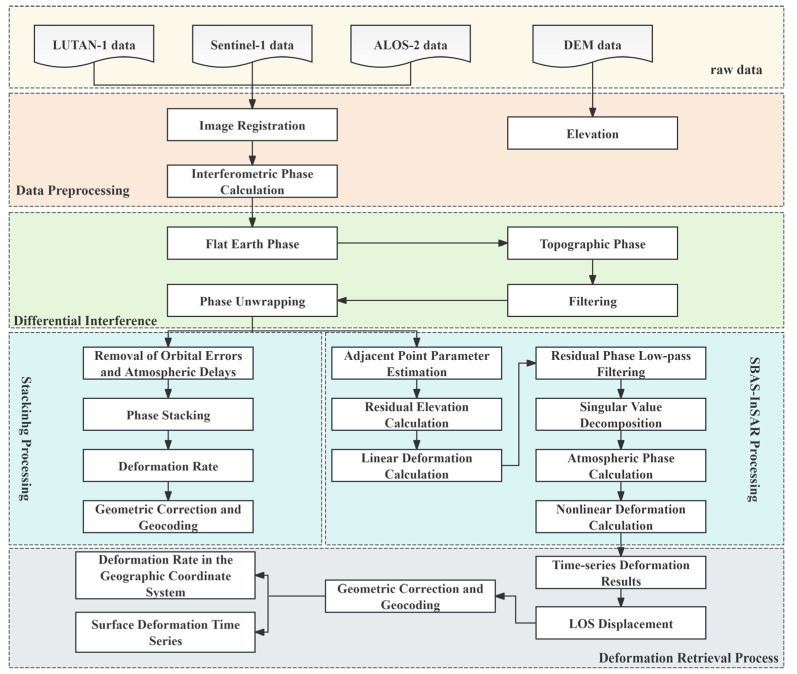
Workflow of SAR data processing.

The LT-1 data processing pipeline comprised the following three steps: First, the interferometric pairs were generated by selecting adjacent SAR acquisitions based on spatiotemporal baseline criteria, followed by image coregistration using accurate orbital data. Subsequently, we applied a 5:5 (range: azimuth) multilooking operation to balance resolution and phase quality while employing adaptive Goldstein filtering to enhance the differential interferogram quality. Finally, high-quality pixels were selected through coherence thresholding (γ > 0.3), after which phase unwrapping was performed using the MCF algorithm. The unwrapped phases were then converted to deformation values and geocoded. All processing was implemented using GAMMA2021 software, with the detailed workflow illustrated in Figure 2.

### 3.2. Deformation Results from Different Data and Analysis

#### 3.2.1. Sentinel-1 Results vs. ALOS-2 PALSAR Results

**Figure 3:**
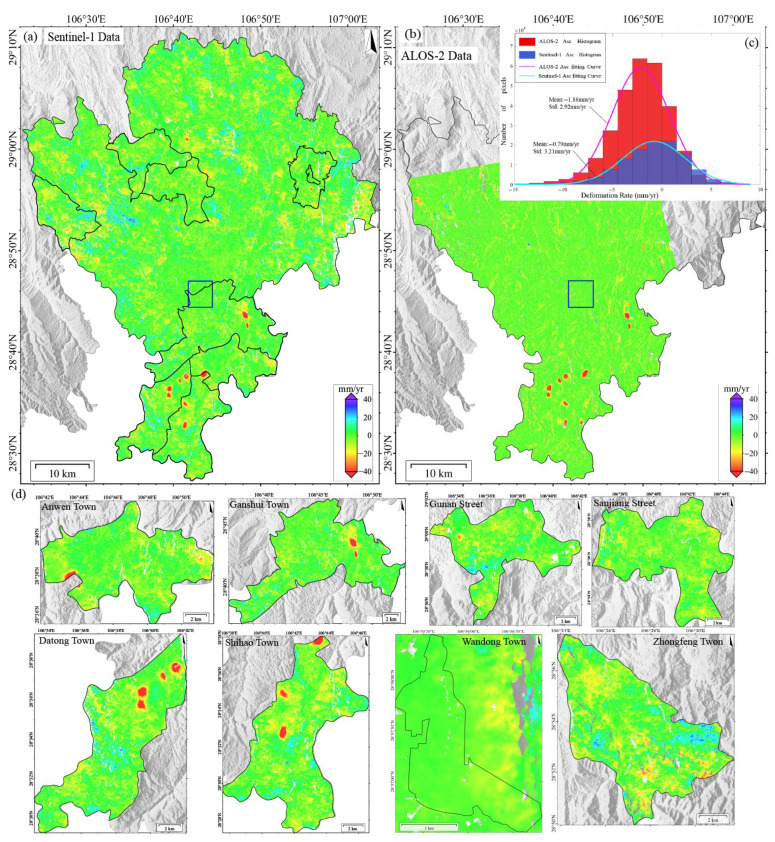
(**a**) Deformation results derived from Sentinel-1 data (2018–2019). (**b**) Deformation results derived from ALOS-2 data (2018–2019). (**c**) Statistical histogram comparing deformation rates between ALOS-2 and Sentinel-1 datasets within the stable reference area (delineated by blue boxes in panels (**a**,**b**)). (**d**) Deformation rates of Anwen Town, Ganshui Town, Gunan Street, Sanjiang Street, Datong Town, Shihao Town, Wandong Town, and Zhongfeng Town (based on Sentinel-1).

The surface deformation in Qijiang, monitored using either Sentinel-1 or ALOS-2 PALSAR data during 2018–2019 (Figure 3), exhibits pronounced spatial heterogeneity. Subsidence centers show strong spatial correlation with coal mining areas. Continuous subsidence zones are concentrated in the southern region (Datong Town–Shihao Town–Anwen Town–Ganshui Town, as shown in Figure 3d), with annual subsidence rates of 1–116 mm/yr (local maximum > 116 mm/yr). In contrast, discrete secondary subsidence zones (1–67 mm/yr, local > 67 mm/yr) are observed in northern areas (Sanjiang Street and Gunan Street). Notably, L-band data (ALOS-2) detected additional deformation signals induced by landslides in Wandong Town.

The deformation rates across the study area follow a skewed distribution, primarily ranging from −20 to +10 mm/yr. However, mining areas exhibit extreme subsidence, with ALOS-2 recording a maximum rate of 140 mm/yr (new subsidence center in Zhongfeng Town), 20.7% higher than Sentinel-1’s maximum (116 mm/yr). This demonstrates L-band’s superior capability for monitoring large-magnitude deformation. Both datasets show high spatial pattern consistency, accurately identifying major subsidence centers. Cross-validation reveals an average boundary positioning deviation of less than 1 pixel (<30 m). To evaluate the monitoring accuracy of Sentinel-1 and ALOS-2 systems, we conducted a histogram analysis of deformation signals within a stable area (blue rectangle in Figure 3a), yielding the following key findings:

Deformation distribution: Both datasets exhibited approximately normal distributions. Results of Sentinel-1 clustered in the -10 to +8 mm/yr range (extremes < 20 mm/yr), and those of ALOS-2 demonstrated a marginally wider distribution (extremes < 25 mm/yr).

Data stability: Both datasets showed mean deformation values approaching zero, indicating excellent baseline consistency.

Monitoring precision: Contrary to theoretical expectations (longer L-band wavelength [23.6 cm] typically increases uncertainty), ALOS-2 exhibited superior precision (standard deviation (STD) = 2.92 mm/yr) than that of Sentinel-1 (3.21 mm/yr). This phenomenon may be due to vegetation canopy effects that may mitigate the L-band’s theoretical limitations. The analysis suggests that environmental factors significantly influence wavelength-dependent performance, and L-band data may improve the monitoring accuracy in mountainous areas.

#### 3.2.2. Sentinel-1 Results vs. LT-1 Results

**Figure 4:**
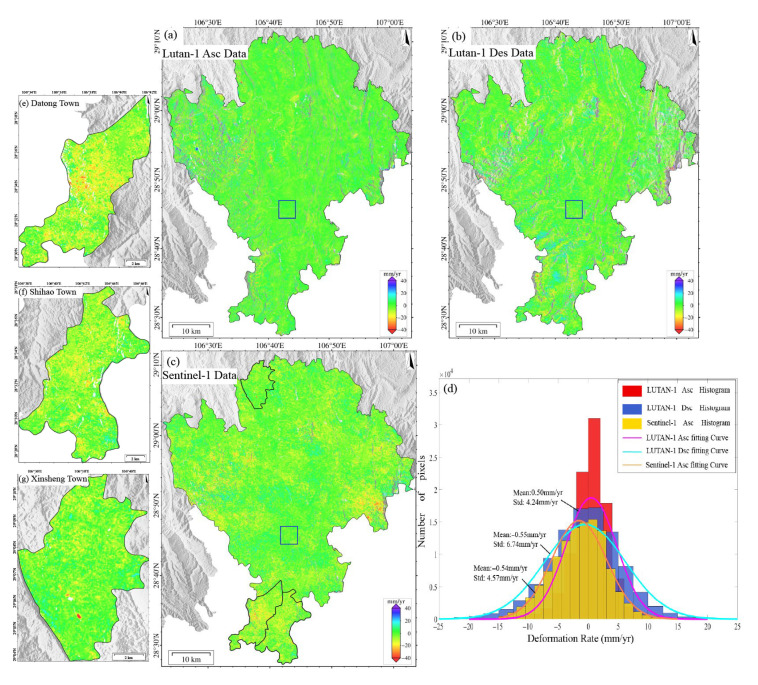
(**a**) Deformation results from LT-1 ascending orbit images (2023–2025). (**b**) Deformation results from LT-1 descending orbit images (2023–2025). (**c**) Deformation results from Sentinel-1 images (2023–2024). (**d**) Statistical histograms of deformation rates for the three datasets within the stable reference area (delineated by blue boxes in panels (**a**–**c**)). (**e**) Deformation results for Datong town (based on Sentinel-1). (**f**) Deformation results for Shihao town (based on Sentinel-1). (**g**) Deformation results for Xinsheng town (based on Sentinel-1).

The surface deformation in Qijiang based on Sentinel-1 (2023–2024) and LT-1 (ascending/descending 2023–2025) is shown in Figure 4. Compared with 2018–2019, the subsidence trend across the region has significantly weakened, with deformation in closed mining areas stabilizing, indicating the initial effectiveness of mining governance measures. The deformation patterns detected by the three datasets are generally consistent. Subsidence primarily occurs in historical mining areas such as Datong Town, Shihao Town, and Xinsheng Town (Figure 4e–g), but the subsidence rates have notably decreased. Sentinel-1 detected deformation rates ranging from −87 to +42 mm/yr, with localized extreme subsidence exceeding 87 mm/yr. LT-1 ascending data show a dominant deformation range of −25 to +20 mm/yr, with localized extremes exceeding 300 mm/yr. LT-1 descending data exhibit a dominant deformation range of −30 to +30 mm/yr, with localized extremes surpassing 400 mm/yr. These discrepancies primarily arise from the stronger penetration capability of the L-band (23.8 cm) compared to the C-band (5.6 cm), which effectively mitigates vegetation decorrelation effects and thus captures more substantial true deformation.

We selected a stable area (blue box in Figure 4a) for statistical analysis of the deformation histograms (Figure 4d). The deformation rates from all three datasets follow a normal distribution (>85% within −10 to +10 mm/yr), with mean values of −0.54 mm/yr (Sentinel-1), 0.50 mm/yr (LT-1 ascending), and −0.55 mm/yr (LT-1 descending), indicating negligible systematic bias. The LT-1 ascending data exhibit the smallest STD (4.24 mm/yr), outperforming Sentinel-1 (4.57 mm/yr) and the descending data (6.74 mm/yr). The superior precision of the LT-1 ascending data is attributed to greater data availability and reduced topographic distortion effects due to favorable observation geometry. In SMRC, the L-band LT-1, with sufficient data coverage, provides higher monitoring accuracy than the C-band (Sentinel-1) data, particularly in vegetated areas and large-gradient deformation scenarios.

## 4. Impact of Sensors and Observation Geometry on Landslide Monitoring Capability

**Figure 5:**
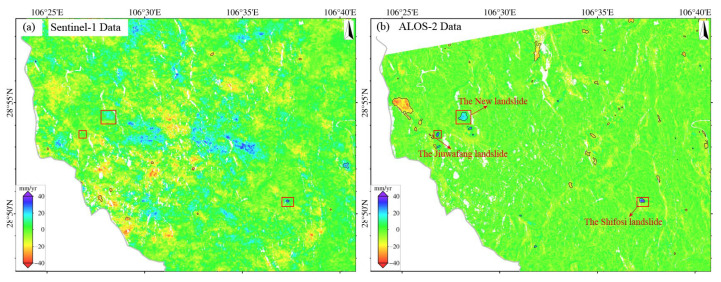
Three typical landslide identification results. (**a**) Sentinel-1 results. (**b**) ALOS-2 results. The three landslides are obvious in ALOS-2 results, but only the Shifosi landslide is identified in the Sentinel-1 results.

To investigate the influence of different sensors and observation geometries on InSAR-based landslide monitoring, we selected three typical landslides in the study area (as shown in Figure 5), including two landslides in Zhongfeng Town (the Jiuwafang and the New landslides) and the Shifosi landslide in Guofu Town. We compared the deformation velocity maps and landslide boundary discrepancies derived from Sentinel-1, ALOS-2, and LT-1 to investigate how sensor specifications and acquisition geometries affect InSAR landslide detection.

The Jiuwafang landslide is located in Zhongfeng Village, Qijiang District, on the northern flank of the Jinhua Mountain incline, which is a downslope with a slope angle of about 21°. It covers an area of 40.5 × 10^4^ m^2^ and has an estimated volume of 283.5 × 10^4^ m^3^. Historical records indicate no obvious sign of deformation in the area, but since 2020, movement has been observed, including tensile cracks along a road at the landslide front and cracks in buildings and farmland, accompanied by localized sliding, near the rear scarp.

The Shifosi landslide, located in Gusong Village, Qijiang District, is a large ancient rocky landslide that has experienced slow, continuous movement since 2000. It lies on the west flank of Zhuantang backslope with an inclination of about 12°, a length of about 1300 m, a width of 300 m, and a thickness of 18 m, covering 39.0 × 10^4^ m^2^, and with a volume of about 702.0 × 10^4^ m^3^. Deformation is dominated by creeping sliding, with fissures concentrating in the frontal zone, and localized deflections/uplifts influenced by micro-geomorphology.

### 4.1. Performance Evaluation of Sentinel-1 and ALOS-2 for Landslide Monitoring

**Figure 6:**
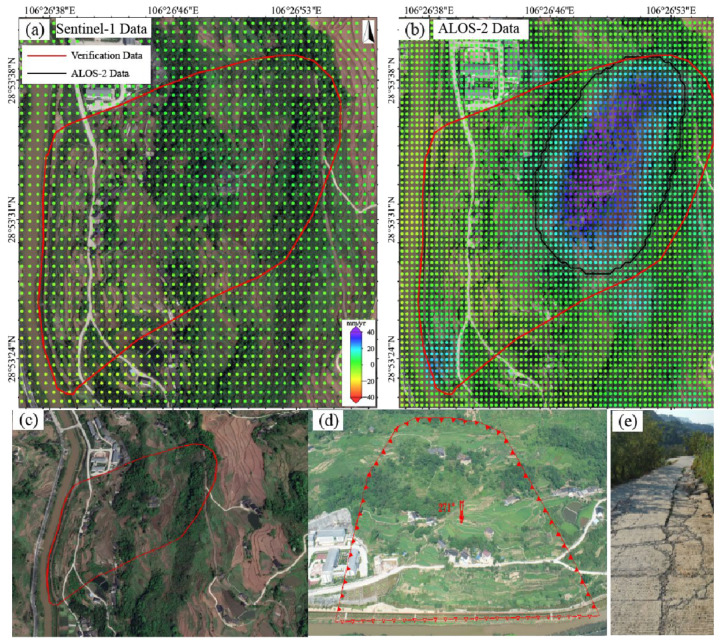
The Jiuwafang landslide. (**a**) Deformation velocity map derived from Sentinel-1 data. (**b**) Deformation velocity map derived from ALOS-2 data. (**c**) Optical image of the landslide. (**d**) Field validation photo showing the whole landslide. (**e**) Road cracks induced by landslide deformation.

**Figure 7:**
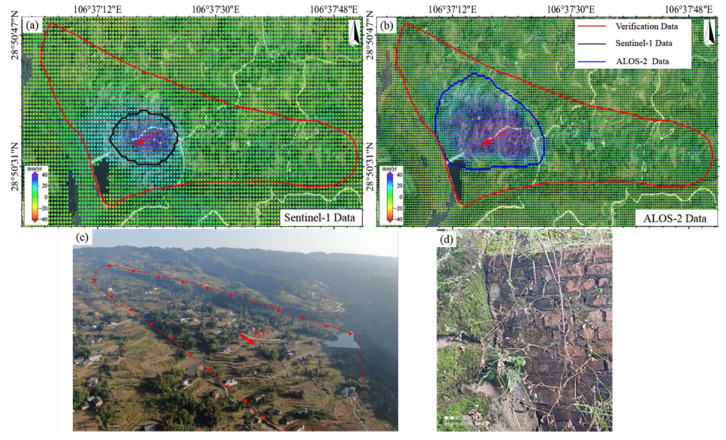
The Shifosi landslide (The red arrows indicate the main direction of landslide movement). (**a**) Deformation velocity map derived from Sentinel-1 data. (**b**) Deformation velocity map derived from ALOS-2 data. (**c**) Field validation photo showing the whole landslide. (**d**) Wall cracks caused by the landslide deformation.

**Figure 8:**
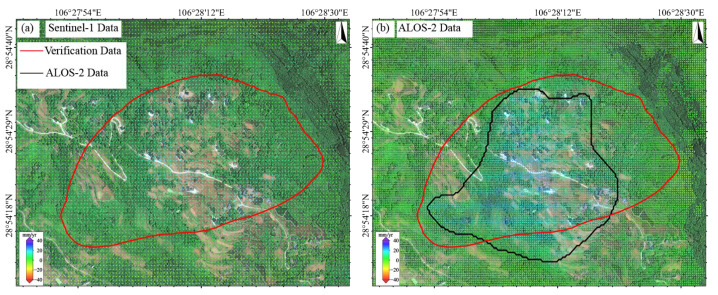
The New landslide. (**a**) Deformation velocity map derived from Sentinel-1 data. (**b**) Deformation velocity map derived from ALOS-2 data.

At the Jiuwafang landslide (Figure 6), ALOS-2 identified a deformation zone in the central sector (maximum rate > 30 mm/yr), spatially consistent with field-observed road cracks (Figure 6e). In contrast, the Sentinel-1 result showed no significant deformation features. At the Shifosi landslide (Figure 7), both datasets detected deformation but with notable discrepancies. In terms of magnitude, ALOS-2 recorded values of −12.3 to 46.9 mm/yr, ~40% higher on average than Sentinel-1 (−7.9 to 34.8 mm/yr). Spatially, ALOS-2 covered 25% more area than Sentinel-1 and exhibited better alignment with the actual landslide boundary (offset < 15 m). For the New landslide (Figure 8), ALOS-2 was the only sensor that detected subtle deformation signals, with its identified extent closely matching the actual landslide boundary. In contrast, Sentinel-1 failed to capture any noticeable deformation features.

Analysis shows that these differences stem from the following three factors: resolution, wavelength, and topographic adaptability. ALOS-2 (3 × 3 m) resolves small-area landslide features more precisely than Sentinel-1 (5 × 20 m). The L-band (23.6 cm) outperforms the C-band (5.6 cm) in vegetated areas due to superior penetration and low sensitivity to decorrelation. Sentinel-1 is more prone to geometric distortions in steep terrain (mean slope > 25°).

In heavily vegetated regions in SMRC, ALOS-2 data surpass Sentinel-1 in landslide detection accuracy, deformation magnitude retrieval, and spatial coverage. The ALOS-2 data are particularly suitable for early identification of small to medium landslides. These findings provide critical guidance for sensor selection in geohazard-prone areas.

### 4.2. Performance Evaluation of LT-1 and Sentinel-1 for Landslide Monitoring

**Figure 9:**
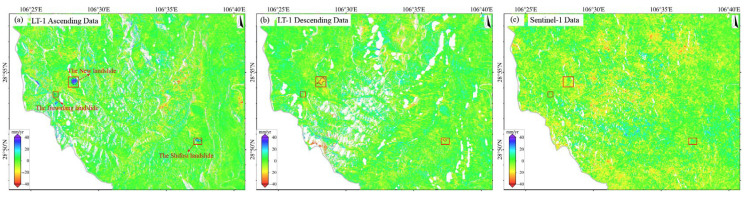
Three typical landslide identification results. from (**a**) LUTAN-1 ascending data, (**b**) LUTAN-1 descending data and (**c**) Sentinel-1 data.

**Figure 10:**
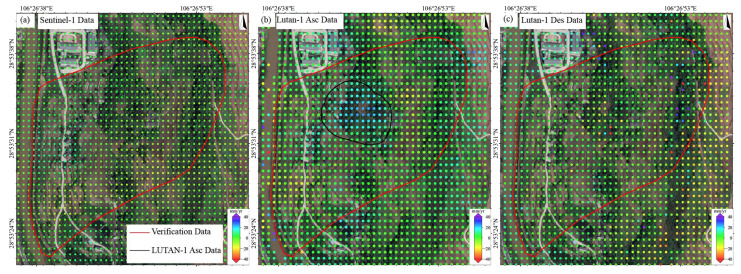
The Jiuwafang landslide. Deformation velocity map derived from (**a**) Sentinel-1 data, (**b**) LT-1 ascending data, and (**c**) LT-1 descending data.

Based on the multi- temporal InSAR (2023/06–2025/01) data, we systematically compared the performance of the LT-1 and Sentinel-1 data in landslide monitoring. The LT-1 ascending data successfully detected the Jiuwafang, Shifosi, and New landslides, while the descending data only captured the latter two. In contrast, Sentinel-1 failed to identify significant deformation signals at all three sites (Figure 9). For the Jiuwafang landslide (Figure 10), only the LT-1 ascending data revealed localized, small-magnitude deformation. This performance difference can be attributed to two main factors. First, the Jiuwafang landslide has been gradually stabilizing over time, exhibiting annually decreasing deformation rates. Second, the ascending data is more sensitive to the slope’s displacement direction, compared to the descending geometry. Additionally, the lower spatial resolution and coherence performance of Sentinel-1 limited its ability to detect such small-scale, slow-moving (creeping) landslides.

**Figure 11:**
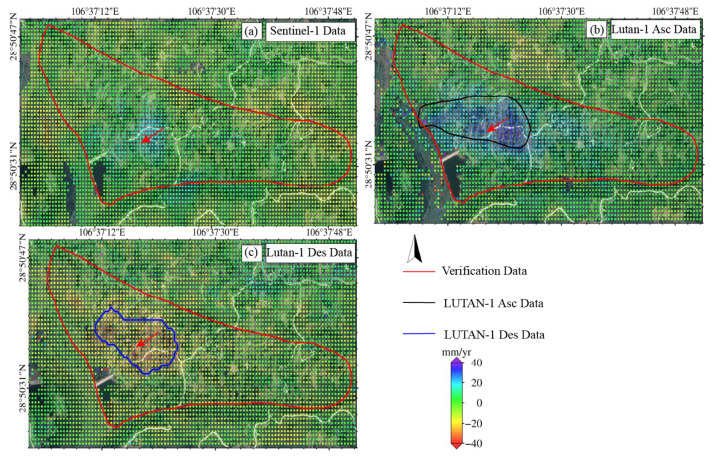
The Shifosi landslide (The red arrows indicate the main direction of landslide movement). Deformation velocity map derived from (**a**) Sentinel-1 data, (**b**) LT-1 ascending data, and (**c**) LT-1 descending data. The red arrows indicate the direction of the landslide.

**Figure 12:**
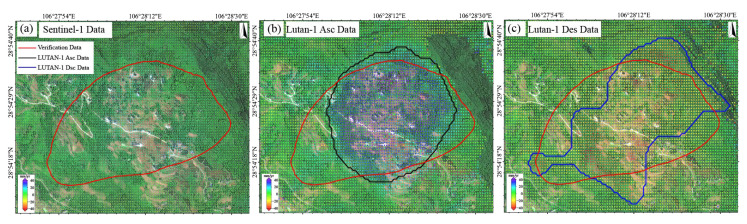
The New landslide. Deformation velocity map derived from (**a**) Sentinel-1 data, (**b**) LT-1 ascending data, and (**c**) LT-1 descending data.

For the Shifosi landslide (Figure 11), the LT-1 ascending data covered 35% more area and detected larger deformation magnitude (−9.9 to 66 mm/yr) than descending data (−36 to 0.9 mm/yr). This disparity arose because the landslide’s main movement direction aligned more closely with the ascending LOS, enhancing its sensitivity to deformation. Time-series analysis (Figure 12) showed deformation rates of +65 to −25 mm/yr (LT-1 ascending) and +47 to −55 mm/yr (LT-1 descending) for the New landslide. Compared to ALOS-2 results (2018–2019), these rates increased by 30–40%, indicating accelerated deformation.

These findings suggest that LT-1 outperforms Sentinel-1 in detecting small to medium landslides in SMRC. In addition, multi-geometry observations mitigate limitations of single-track data, improving spatial coverage and deformation characterization. Therefore, a multi-sensor, multi-geometry fusion strategy is critical for accurate landslide monitoring in complex terrains.

### 4.3. Comparison of Landslide Recognition Capabilities of InSAR in Different Bands

To systematically evaluate the performance of different InSAR bands for landslide identification in complex terrain, we conducted a comparative study using L-band ALOS-2/PALSAR-2 and C-band Sentinel-1 data in 2018–2019. The Qijiang District is characterized by steep terrain, frequent geological hazards, and a complex geological setting. The northern-central part of the district features a typical inverted mountain, hazardous rock belt pregnant area, while the central part is a monoclinic mountain, smooth rocky landslide pregnant area, with a complex geological environment. These conditions make an ideal testbed for assessing the applicability of the InSAR technology in such environments.

**Figure 13:**
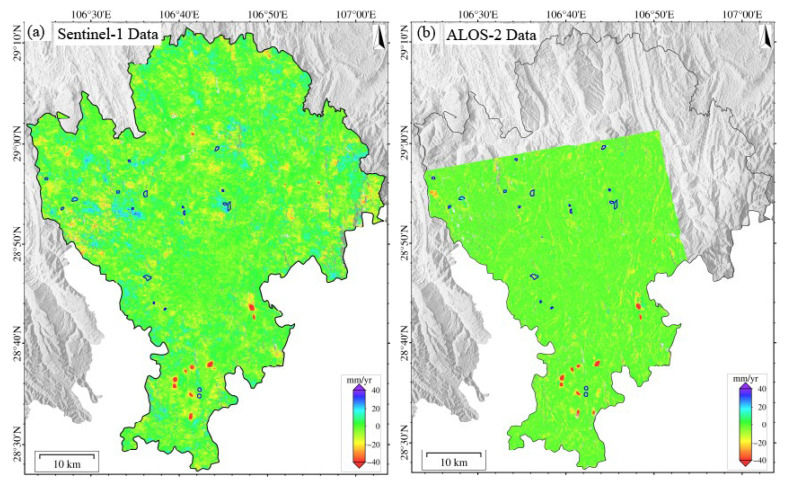
Spatial distribution of geohazards newly identified based on (**a**) Sentinel-1 deformation results and (**b**) ALOS-2/PALSAR deformation results.

A total of 80 potential landslide hazards were identified in this study [27]. Field verification confirmed 18 as new geological hazards, 24 as existing hazards, and 38 as non-geological hazards. Statistical analysis showed that ALOS-2/PALSAR-2 (L-band) successfully identified 14 new landslides (77.8%), significantly outperforming Sentinel-1 (C-band), which identified only 5 (27.8%) (Table 2, Figure 13).

This difference is mainly due to two factors. First, ALOS-2’s finer 3 m resolution (versus Sentinel-1’s 5 × 20 m) enables clearer detection of small-scale landslide deformation features. Second, the L-band’s longer wavelength (~23.6 cm) provides superior penetration through vegetation compared to the C-band (~5.6 cm), mitigating coherence loss in densely vegetated areas like the Qijiang District. For example, in the paragneiss landslide zone in the central part of the study area, Sentinel-1 data exhibited severe coherence degradation during summer due to vegetation growth, resulting in complete deformation signal loss. In contrast, the ALOS-2 data maintained a stable interferometric phase quality.

This study demonstrates that L-band InSAR offers significant advantages for landslide identification in the complex environment of SMRC. With its high spatial resolution and long wavelength, L-band data not only improve the detection of small landslides but also enhance performance in steep terrain and dense vegetation areas. These findings provide an important basis for the selection of sensors in geohazard-prone areas, strongly supporting the preferential use of L-band data for InSAR monitoring in similar areas.

## 5. Discussion

### 5.1. Improved Landslide Detection and Monitoring in SMRC Using LT-1 Data

Compared to Sentinel-1 (wavelength: 5.6 cm) data, LT-1′s (L-band wavelength: 24 cm) data demonstrate superior deformation monitoring performance in the complex terrain of SMRC. The longer wavelength provides stronger penetration capability, effectively penetrating both dense vegetation canopies and shallow loose deposits to detect deformation signals closer to the true ground surface. This advantage is clearly evidenced by the monitoring results from the Jiuwafang, New, and Shifosi landslides. Additionally, the L-band exhibits lower sensitivity to atmospheric delays, resulting in reduced phase noise and consequently higher accuracy in deformation inversion.

LT-1 further features a higher incidence angle range (35–45°), which is advantageous in the SMRC with its rugged topography and variable slope orientations. This design reduces geometric distortions while achieving a higher slant-range spatial resolution (3 m) compared to Sentinel-1′s 5 m resolution. The enhanced resolution enables more precise detection of subtle deformation features associated with small-area landslides. Meanwhile, the L-band is less sensitive to seasonal variations in vegetation and can maintain relatively high coherence even during the rainy season, whereas the C-band experiences a more significant decline in coherence under the same conditions [28]. These advantages enable LT-1 to consistently monitor slow deformation signals in highly vegetated slopes, while Sentinel-1 frequently suffers from data gaps in such areas [29,30].

In this paper, the same verification area was selected for an accurate comparison of ALOS-2 and LT-1 data. As can be seen from Figure 3c and Figure 4d, the standard deviations of the ascending/descending orbits of ALOS-2 and LT-1 are 2.92 mm/yr, 4.24 mm/yr, and 6.74 mm/yr. The higher precision and lower dispersion of ALOS-2 results can be attributed to its superior system maturity, advanced radar performance, and more precise orbit/attitude control. However, ALOS-2 faces limitations, including sparse data availability, high acquisition costs, and only ascending-track coverage in SMRC. In contrast, LT-1 offers greater data accessibility and dual-geometry observations, which enable synergistic monitoring, overcoming blind zones inherent to single-track systems. Combined ascending/descending inversions enhance deformation characterization accuracy. This synergistic observation strategy can mitigate the limitations caused by the misalignment between the LOS direction and the primary direction of landslide movement, thereby improving the overall spatial coverage and reliability of landslide detection.

Thus, LT-1 provides a practical advantage for long-term landslide monitoring in SMRC, delivering reliable data support for efficient landslide identification and dynamic tracking in complex terrains.

### 5.2. Advantages of Multi-Source SAR Data Fusion for Landslide Identification in SMRC

The integration of multi-source SAR data can significantly enhance landslide detection and monitoring capabilities in SMRC. C-band SAR (e.g., Sentinel-1) offers high temporal resolution (6- to 12-day revisit cycle), making it suitable for capturing rapid, shallow landslides triggered by heavy rainfall. In contrast, L-band SAR (e.g., LT-1, ALOS-2) exhibits superior penetration capability, maintaining high coherence in vegetated areas, and is thus more effective for monitoring deep-seated, slow-moving landslides. Combining deformation results from both bands enables cross-validation, improving reliability and reducing false detection. For example, Chen et al. [31] identified 120 potential landslides using C-band data alone in a landslide-prone area of western Hunan, but an additional 35 deep-seated landslides were detected after incorporating L-band data, a 30% increase in identification rate. Moreover, multi-source SAR data fusion improves temporal resolution, enhancing landslide deformation capture and enabling timely detection of active slope movements. In this study, the acquisition of ALOS-2 data is temporally uneven, with significant observation gaps. Relying solely on ALOS-2 data therefore imposes limitations on the continuous monitoring of landslide dynamics. Integrating data from multiple SAR sensors enhances both the spatial coverage and temporal continuity of landslide monitoring, thereby improving the accuracy and comprehensiveness of geological hazard risk assessments.

While C-band SAR suffers from signal attenuation in dense vegetation, it excels in urban and engineered environments. Its shorter wavelength (5.6 cm) enables millimeter-level sensitivity to subtle deformations (e.g., building subsidence, road cracks), making it ideal for monitoring urban geohazards (e.g., ground subsidence, slope failure). Sentinel-1 successfully detected minor deformations induced by construction activities in a southwestern town [32]. However, L-band data, due to its longer wavelength, often missed such shallow, small-scale movements. In addition, urban areas provide abundant persistent scatterers (e.g., buildings and bridges), enhancing the reliability of C-band-based time-series InSAR techniques (e.g., PS-InSAR, SBAS-InSAR).

In conclusion, the integration of multi-source SAR data creates a synergistic monitoring framework, where the C-band effectively compensates for the L-band’s limitations in urban and human-modified areas, and the L-band overcomes the C-band’s deficiencies in vegetated regions. This complementary relationship significantly enhances InSAR technology’s adaptability and identification accuracy for various landslide types across diverse terrains and environments. The combined approach provides more precise technical support and a robust data foundation for comprehensive prevention and control of complex geological hazards in SMRC.

However, the current lack of multi-source SAR datasets with consistent temporal and spatial resolutions in the study area prevents systematic evaluation of multi-sensor and multi-geometry SAR data for geohazard monitoring and identification under complex vegetation coverage. With the continuous accumulation and increasing availability of multi-source SAR data, particularly from C- and L-band sensors, future studies can be conducted on the influence of vegetation type, coverage density, and seasonal variation on monitoring performance. This will improve the stability and applicability of InSAR technology in densely vegetated, humid areas.

## 6. Conclusions

This study systematically evaluated landslide-prone areas in the Qijiang District of Chongqing, using multi-source SAR data to assess sensor- and geometry-dependent variations in landslide detection. Key findings include the following: (1) Sentinel-1 (C-band) excelled in detecting shallow deformations in urban/mining areas, while ALOS-2 and LT-1 (L-band) outperformed in vegetated and complex terrains, proving more effective for small to medium landslides. (2) The LT-1 ascending/descending data comparison revealed complementary coverage, with ascending orbits exhibiting less distortion and better detection, while descending orbits improved spatiotemporal continuity. (3) Integrating multi-band, multi-geometry SAR data increased spatiotemporal resolution, reduced false/missed detections, and improved the identification of slow-moving and deep-seated landslides. This work demonstrates the synergistic advantages of multi-source SAR for landslide monitoring in the Qijiang District, providing a reference for SAR-based hazard assessment and supporting landslide risk mitigation in SMRC.

## Figures and Tables

**Table 1:** Parameters of the SAR data used in this study.

Parameters	Sentinel-1	ALOS-2 PALSAR	LUTAN-1	LUTAN-1
flight direction	ascending orbit	ascending orbit	ascending orbit	descending orbit
wavelength	5.6 cm (C-band)	23.6 cm (L-band)	23.6 cm (L-band)	23.6 cm (L-band)
theoretical revisit cycle	6/12	14	8/4	8/4
incidence (°)	44.2~44.3	39.6~39.7	10~60	10~60
number of images	58/44	9	54	60
acquisition time	201801~201912; 202301~202411	201804~201912	202304~202501	202306~202501

**Table 2:** List of information on new geohazard sites.

Ordinal Number	Hidden Danger Point Number	Results of Verification	ALOS2	Sentinel-1	$\mathrm{Area}\;(\text{×}10^{4}{\;m}^{2})$
1	2208QIJ012	additional landslide	√	√	4.9
2	2208QIJ007	additional landslide	√	×	7.5
3	2208QIJ008	additional landslide	√	×	8.52
4	2208QIJ031	additional landslide	√	×	9.1
5	2208QIJ032	additional landslide	√	×	9.1
6	2208QIJ029	additional landslide	√	×	10.73
7	2208QIJ022	additional landslide	√	×	13.8
8	2208QIJ049	additional landslide	√	√	17.7
9	2208QIJ019	additional landslide	√	×	18.5
10	2208QIJ030	additional landslide	√	×	25
11	2208QIJ042	additional landslide	×	√	32.7
12	2208QIJ041	additional landslide	×	√	36.1
13	2208QIJ001	additional landslide	√	×	40.5
14	2208QIJ016	additional landslide	√	×	50.83
15	2208QIJ026	additional landslide	√	×	53.55
16	2208QIJ035	additional landslide	√	×	56.3
17	2208QIJ034	additional landslide	×	×	71.7
18	2208QIJ050	additional landslide	×	√	84

## Data Availability

The Sentinel-1 SAR data used in this study are copyrighted by the European Space Agency (https://dataspace.copernicus.eu, accessed on 1 March 2022).
